# Use of open-ended Foley catheter to treat profuse urine leakage around suprapubic catheter in a female patient with spina bifida who had undergone closure of urethra and suprapubic cystostomy: a case report

**DOI:** 10.4076/1757-1626-2-6851

**Published:** 2009-06-12

**Authors:** Subramanian Vaidyanathan, Bakul M Soni, Peter L Hughes, Gurpreet Singh

**Affiliations:** 1 Regional Spinal Injuries Centre, District General HospitalSouthport PR8 6PNUK; 2 Department of Radiology, District General HospitalSouthport PR8 6PNUK; 3 Department of Urology, District General HospitalSouthport PR8 6PNUK

## Abstract

**Introduction:**

Leakage of urine around a catheter is not uncommon in spinal cord injury patients, who have indwelling urethral catheter. Aetiological factors for leakage of urine around a catheter are bladder spasms, partial blockage of catheter, constipation, and urine infection. Usually, leakage of urine subsides when the underlying cause is treated. Leakage of urine around a suprapubic catheter is very rare and occurs in patients, in whom the urethra is closed due to severe stricture or previous surgery.

**Case presentation:**

We describe a 35-year-old female patient with spina bifida and paraplegia, who had undergone suprapubic cystotomy followed by urethral closure for leakage of urine per urethra. She developed leakage of urine around suprapubic Foley catheter, which did not subside even after changing the catheter, ruling out vesical calculus, and ensuring that there was no kink in catheter or drainage tube. As a desperate measure, we punched a large hole at the tip of a Foley catheter and used this catheter for suprapubic drainage. Leakage of urine around suprapubic catheter stopped and the patient was greatly relieved.

**Conclusion:**

Leakage of urine around a catheter requires prompt attention in spinal cord injury patients; otherwise patients can develop maceration of neuropathic skin and pressure sore. Management of spinal cord injury patients with leakage of urine around a suprapubic catheter should include (i) changing the catheter, (ii) prescribing anticholinergic drugs to control bladder spasm, (iii) treating constipation and urine infection when present, (iv) imaging studies or flexible cystoscopy to look for vesical calculus. If leakage of urine persists despite all these measures, use of a modified Foley catheter in which, a large hole has been made at the tip, is worth trying.

## Introduction

People with spinal cord injury, who have indwelling urethral catheter drainage, experience occasional leakage of urine around the catheter. Common aetiological factors for leakage of urine around a catheter are: partially blocked catheter, bladder spasms, constipation or impacted stool, urine infection, and vesical calculus. Sometimes, there may be a kink in the catheter or drainage tubing. Flushing the catheter with sterile 0.9% sodium chloride solution may unblock a catheter. Bladder washouts are not indicated in spinal cord injury patients, who exhibit autonomic dysreflexia.

In contrast to leakage of urine around urethral catheter, leakage of urine around suprapubic catheter is very rare. Leakage of urine around suprapubic catheter occurs in patients, who have severe stricture of urethra or undergone surgery for closure of urethra. We present a female patient with spina bifida and paraplegia, who underwent suprapubic cystostomy and then closure of urethra for leakage of urine from urethra. Subsequently, she was all right for a few years but developed profuse leakage of urine around suprapubic catheter. As a desperate measure, we punched a large hole at the tip of a Foley catheter and used this catheter for suprapubic drainage. Leakage of urine around suprapubic catheter stopped and the patient was greatly relieved.

## Case presentation

**Figure 1.**
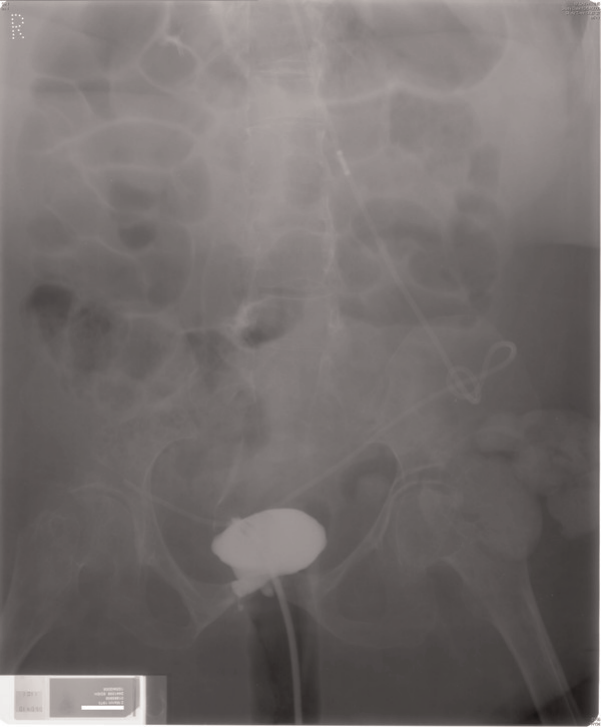
Cystogram, performed on 10 April 2006, showed urinary bladder of small capacity. Closure of urethra had been performed; therefore, only few millimetres of proximal urethra are opacified by the contrast.

This Caucasian lady was born in 1973 with spina bifida and paraplegia. Her mother had been looking after her very well. This patient managed neuropathic bladder by pads until the age of twelve years. Subsequently she had indwelling urethral catheter drainage. In the year 2000, this lady was prescribed diuretic as she had developed swelling of both lower extremities. After taking diuretics, she developed leakage of urine around the catheter (bypassing). She got fed up especially because of the smell of urine. She continued to experience severe bypassing of catheter despite taking tolterodine 2 milligrams, three times a day. In 2001, suprapubic cystostomy was performed in order to cure leakage of urine around urethral catheter. Even after undergoing suprapubic cystostomy, this lady continued to get problems with suprapubic catheter. Some days, she was completely dry; but some days, there would not be any drainage in suprapubic catheter. In May 2002, clinical examination revealed that the urethra was wide open as before, and the tip of suprapubic catheter was protruding in to urethra. She was advised to have a Foley catheter with only five ml of water for inflation of balloon. She continued to experience leakage of urine per urethra. She had developed pressure sores in perineal and ischial region, which were made worse by urine leakage per urethra. She wore pads in addition to suprapubic catheter. In September 2005, perineal urethral closure was performed. After undergoing closure of urethra, she did not have leakage of urine per urethra. A follow-up cystogram, performed in 2006, showed a small capacity bladder. There was no leakage of contrast from urethra (Figure 1).

This patient had been taking modified-release oxybutynin 20 milligrams a day. Other medications were folic acid and docusate sodium now and again. Suprapubic catheter drained urine satisfactorily until 2009 when this patient developed leakage of urine around suprapubic catheter. Suprapubic catheter was changed after 15 days, then 7 days, and then after three days. Persistent leakage of urine resulted in soggy skin of inner thighs and buttocks. Understandably, both the patient and patient's mother were concerned that skin might break down and she might develop pressure sores.

**Figure 2.**
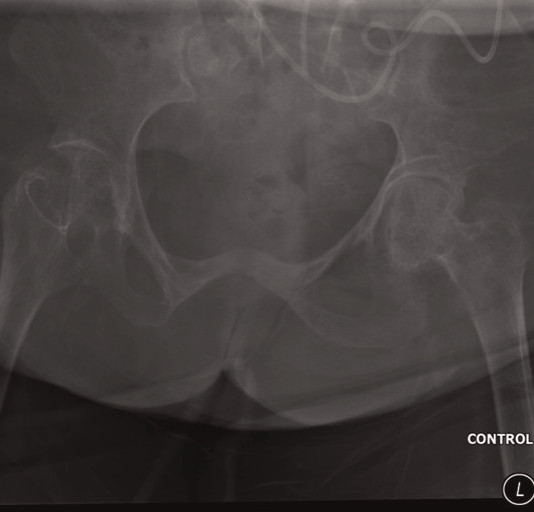
Control film, taken on 06 March 2009, showed no stone in urinary bladder.

**Figure 3.**
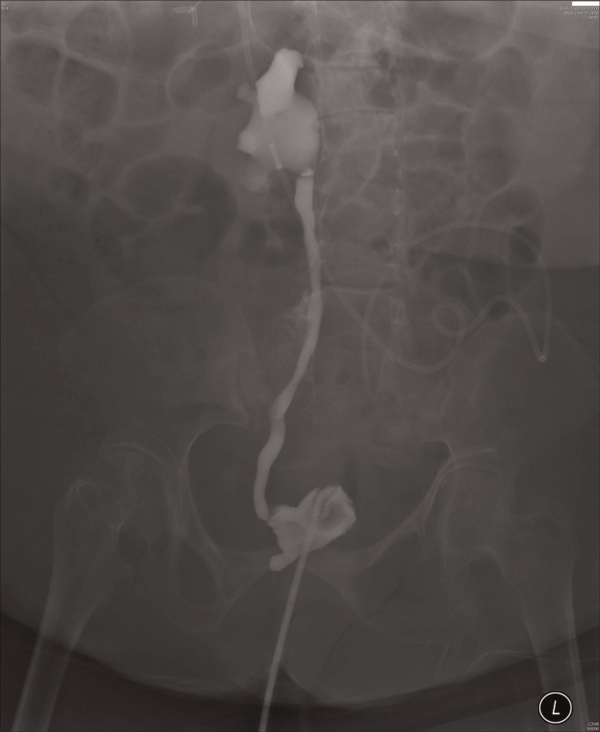
Cystogram, performed on 06 March 2009, showed small capacity bladder and grade IV vesicoureteric reflux on right side.

**Figure 4.**
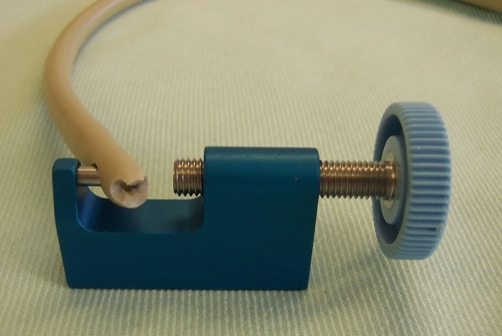
Photograph of a Foley catheter in which a large hole has been made at the tip by using a Foley catheter tip Punch three times, each hole, overlapping the previous one.

**Figure 5.**
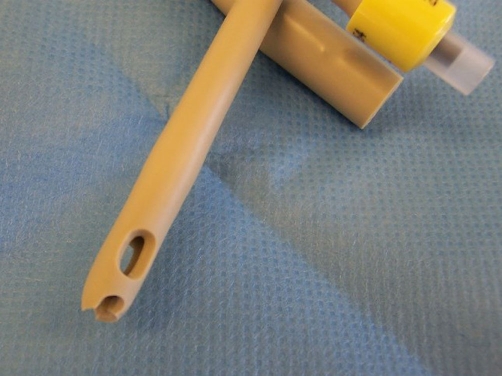
Close-up view of Foley catheter with a large, rugged hole at the tip.

X-ray of urinary bladder showed no stone in urinary bladder (Figure 2). Cystogram revealed very small capacity bladder with grade 4 vesicoureteric reflux on right side (Figure 3). As a desperate measure, we made a large hole at the tip of a size 20 French, Bard Biocath Foley catheter, by using a Foley catheter tip punch three times, each hole overlapping the previous one (Figure 4). This resulted in a large hole at the tip of Foley catheter, which was then inserted for suprapubic drainage. (Figure 5) Leakage around the catheter stopped. The patient and her mother were delighted.

## Discussion

Leakage of urine around urethral catheter is not uncommon and varies with the type of catheter. The incidence of bypassing was 28% in the Biocath group and 52.8% in the Silastic group [1]. When a patient develops leakage of urine around a urethral Foley catheter, use of a Pemberton catheter, which has a hole immediately below the Foley balloon, is indicated to achieve complete drainage of urine from urinary bladder [2]. But in a patient, who uses suprapubic catheter for drainage of particularly small bladder, use of an open ended Foley catheter is likely to result in improved drainage of urine from the bladder. Open-ended all-silicone Foley catheters are commercially available, but drainage hole in these catheters is usually small. Another disadvantage of using an all-silicone Foley catheter is cuffing after deflation and consequent difficulty in removing a deflated catheter. Suprapubic profilometry confirmed increased resistance to withdrawal by formation of a ‘cuff’ on deflation of the balloon of all-silicone catheters. Therefore, Parkin and associates [3] recommended that the first choice of catheter material for long-term urethral and suprapubic use should be hydrogel-coated latex. A search of Cochrane Reviews revealed one clinical trial, which suggested that the use of a hydrogel coated latex catheter rather than a silicone catheter might be better tolerated (RR for need for early removal 0.41, 95% CI 0.22 to 0.77) [4].

**Figure 6.**
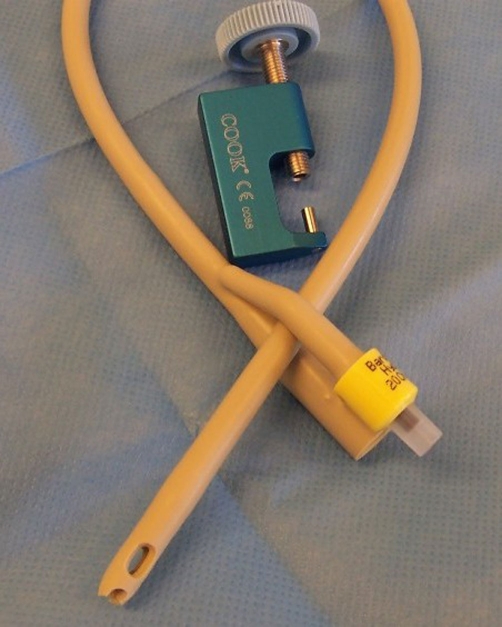
Photograph of Foley catheter tip Punch and hydrogel coated Foley catheter, which are required for changing suprapubic catheter in this patient.

Foley catheter tip punch (Figure 6) is designed for cutting 0.118 inch (3.0 mm) diameter round hole in the distal end of a Foley catheter, 14 French or larger. [5] We created a large hole at the tip of a 20 French Bard Biocath Foley catheter [6] by using a Foley catheter tip Punch three times, each hole, overlapping the previous one. This catheter with a large hole at the tip of Foley catheter (Figure 5) was then inserted for suprapubic drainage in our patient. Leakage around the catheter stopped. The patient and her mother were delighted.

The authors admit that despite a thorough search for evidence relating to the effectiveness of different catheter policies for adults with neurogenic bladder, no evidence was found in the Cochrane Reviews [7]. Similarly, no eligible trials were identified that compared alternative routes of catheter insertion. [8] Further, there was no consensus regarding the indications for use of catheter wash-outs nor the method of administration, frequency, duration of administration and choice of solution [9]. Our case shows that use of open-ended Foley catheter may be useful in some patients with neuropathic bladder, who experience persistent catheter bypassing. For ethical reasons, we did not use ordinary Foley catheter subsequently in this patient to find out whether catheter bypassing would recur if the type of catheter were changed from open-ended Foley catheter to ordinary Foley catheter.

## Conclusion

Leakage of urine around a catheter requires prompt attention in spinal cord injury patients; otherwise patients can develop maceration of neuropathic skin and pressure sore. Management of spinal cord injury patients with leakage of urine around a suprapubic catheter should include (i) changing the catheter, (ii) prescribing anticholinergic drugs to control bladder spasm, (iii) treating constipation and urine infection when present, (iv) imaging studies or flexible cystoscopy to look for vesical calculus. If leakage of urine persists despite all these measures, use of a modified Foley catheter in which, a large hole has been made at the tip, is worth trying.

## List of abbreviations

CI: Confidence interval
RR: Relative risk
